# Efficacy of artemether-lumefantrine in relation to drug exposure in children with and without severe acute malnutrition: an open comparative intervention study in Mali and Niger

**DOI:** 10.1186/s12916-016-0716-1

**Published:** 2016-10-24

**Authors:** Lise Denoeud-Ndam, Alassane Dicko, Elisabeth Baudin, Ousmane Guindo, Francesco Grandesso, Halimatou Diawara, Sibiri Sissoko, Koualy Sanogo, Seydou Traoré, Sekouba Keita, Amadou Barry, Martin de Smet, Estrella Lasry, Michiel Smit, Lubbe Wiesner, Karen I. Barnes, Abdoulaye A. Djimde, Philippe J. Guerin, Rebecca F. Grais, Ogobara K. Doumbo, Jean-François Etard

**Affiliations:** 1 Epicentre, Paris, France; 2 Malaria Research and Training Center, Faculté de Médecine et d’Odonto-stomatologie et Faculté de Pharmacie, Université des Sciences Techniques et Technologies de Bamako, Bamako, Mali; 3 Epicentre, Maradi, Niger; 4 Medecins Sans Frontieres, Brussels, Belgium; 5 Medecins Sans Frontieres, New York, NY USA; 6 Division of Clinical Pharmacology, University of Cape Town, Cape Town, South Africa; 7 WorldWide Antimalarial Resistance Network (WWARN), Oxford, UK; 8 Centre for Tropical Medicine and Global Health, Nuffield Department of Medicine, Oxford University, Oxford, UK; 9 TransVIHMI UMI 233, Institut de Recherche pour le Développement (IRD) – Inserm U 1175 – Montpellier 1 University, Montpellier, France

**Keywords:** *Plasmodium falciparum* malaria, Severe acute malnutrition, Artemether-lumefantrine, Treatment outcome, Pharmacokinetics, Niger, Mali

## Abstract

**Background:**

Severe acute malnutrition (SAM) affects almost all organs and has been associated with reduced intestinal absorption of medicines. However, very limited information is available on the pharmacokinetic properties of antimalarial drugs in this vulnerable population. We assessed artemether-lumefantrine (AL) clinical efficacy in children with SAM compared to those without.

**Methods:**

Children under 5 years of age with uncomplicated *P. falciparum* malaria were enrolled between November 2013 and January 2015 in Mali and Niger, one third with uncomplicated SAM and two thirds without. AL was administered under direct observation with a fat intake consisting of ready-to-use therapeutic food (RUTF – Plumpy’Nut®) in SAM children, twice daily during 3 days. Children were followed for 42 days, with PCR-corrected adequate clinical and parasitological response (ACPR) at day 28 as the primary outcome. Lumefantrine concentrations were assessed in a subset of participants at different time points, including systematic measurements on day 7.

**Results:**

A total of 399 children (360 in Mali and 39 in Niger) were enrolled. Children with SAM were younger than their non-SAM counterparts (mean 17 vs. 28 months, *P* < 0.0001). PCR-corrected ACPR was 100 % (95 % CI, 96.8–100 %) in SAM at both day 28 and 42, versus 98.8 % (96.4–99.7 %) at day 28 and 98.3 % (95.6–99.4 %) at day 42 in non-SAM (*P* = 0.236 and 0.168, respectively). Compared to younger children, children older than 21 months experienced more reinfections and SAM was associated with a greater risk of reinfection until day 28 (adjusted hazard ratio = 2.10 (1.04–4.22), *P* = 0.038). Day 7 lumefantrine concentrations were significantly lower in SAM than non-SAM (median 251 vs. 365 ng/mL, *P* = 0.049).

**Conclusions:**

This study shows comparable therapeutic efficacy of AL in children without SAM and in those with SAM when given in combination with RUTF, but a higher risk of reinfection in older children suffering from SAM. This could be associated with poorer exposure to the antimalarials as documented by a lower lumefantrine concentration on day 7.

**Trial registration:**

ClinicalTrials.gov: NCT01958905, registration date: October 7, 2013.

**Electronic supplementary material:**

The online version of this article (doi:10.1186/s12916-016-0716-1) contains supplementary material, which is available to authorized users.

## Background

Malnutrition and *Plasmodium falciparum* malaria frequently coexist in Sahelian countries and account for a large part of under-five morbidity and mortality during their concomitant peak seasons [1, 2].

Malnutrition is associated with a higher risk of infection and infectious episodes contribute to the deterioration of nutritional status [3]. The question of the impact of child malnutrition on malaria susceptibility is still debated, with conflicting results in the literature. However, it is established that children with either acute or chronic malnutrition are at higher risk to develop severe malaria [4], and to die from it [3, 5]. Reciprocally, malaria could favor the occurrence of severe acute malnutrition (SAM), and implementation of malaria preventive strategies have improved the nutritional status of targeted populations [6].

SAM is defined by the anthropometric indicators of weight-for-height z-score (< –3), mid-upper arm circumference (MUAC; < 115 mm), or presence of nutritional edema [7]. SAM may be complicated by the presence of comorbidities which necessitate inpatient treatment. The current recommended World Health Organization standard protocol for assessing antimalarial efficacy excludes children with SAM from the eligible population [8]. Consequently, few studies have assessed the efficacy of antimalarials in SAM children, and were only conducted with the previous generation of antimalarials, i.e., quinine, chloroquine and sulfadoxine-pyrimethamine [9, 10]. Overall, efficacy of these treatments appeared to be reduced, attributed to lower immunity and for quinine and chloroquine to altered pharmacokinetic properties resulting in lower drug concentrations [11, 12].

Although SAM has been associated with increased volume of distribution and intestinal malabsorption of drugs [13, 14], research on the pharmacokinetics and pharmacodynamics of artemisinin-combination therapies (ACTs) in SAM children is currently lacking [15]. Among published efficacy studies, none have measured drug concentrations and more generally, to our knowledge, the pharmacokinetic (PK) properties of ACTs have not been assessed in children with SAM [16, 17].

A recent meta-analysis conducted by the Worldwide Antimalarial Resistance Network (WWARN) indicated that the risk of treatment failure with artemether-lumefantrine (AL) was greatest in children suffering from global malnutrition; however, it did not include SAM children nor did it measure drug concentration [18].

Here, we aim to assess whether the efficacy of AL, the most commonly used ACT, is altered in children with uncomplicated SAM compared to non-SAM children, and to what extent this can be attributed to inadequate drug exposures as reflected by low lumefantrine concentrations. SAM children received ready-to-use therapeutic food (RUTF) concomitantly with their malaria treatment in this intervention study.

## Methods

### Study design and participants

We performed an open comparative intervention study to assess the efficacy of AL and the capillary blood concentrations of lumefantrine in uncomplicated SAM and non-SAM children. The study protocol and procedures have been described elsewhere [19]. The study was conducted in Oulessebougou district hospital, region of Koulikoro, Mali, and the primary healthcare center of Andoume, Maradi city, Niger. In these areas, malaria transmission is hyperendemic with seasonal peaks during the rainy season (between July and November [19]) and AL is recommended as first-line malaria treatment. Each year, during the hunger gap period (generally from June to October), acute malnutrition increases among young children [20, 21]. According to the 2012 Demographic and Health Surveys, the prevalence of global acute malnutrition in the Koulikoro region of Mali (Aug–Sep 2012) and Maradi region of Niger (Jun–Aug 2012) were 8.6 % (95 % confidence interval (CI), 6.7–9.5) and 16.2 % (14.2–18.5), respectively, while those of SAM were 1.8 % (1.0–2.2) and 2.5 % (1.8–3.6), respectively.

Children aged between 6 and 59 months with uncomplicated *P. falciparum* malaria were eligible if they fulfilled criteria listed in Box 1. After their parent or guardian provided written informed consent, children with weight-for-height z-score < –3 or MUAC < 115 mm were enrolled in the “SAM” group, then two children without SAM were subsequently enrolled in the “non-SAM” group. Children with kwashiorkor or complications requiring hospitalization were excluded as were children with severe stunting (height-for-age z-score < –3).

### Procedures

Children were treated with a fixed dose combination of non-dispersible artemether 20 mg-lumefantrine 120 mg (Coartem® Novartis) following the manufacturer weight-based dose recommendation (one tablet per intake for bodyweights < 15 kg; two tablets for those weighing ≥ 15 kg), twice daily for 3 days. The drug was administered under direct observation with a fat intake consisting of milk (one glass, approximately 15 mL), or RUTF (Plumpy’Nut®, one bag of 92 g) in case of SAM. If vomiting occurred within 30 minutes after intake, a second dose was administered. Children vomiting the second dose were given rescue medication (Additional file 1: Table S1) and excluded.

Children were given an insecticide-treated bed net at enrolment. Other treatments included iron and folic acid supplementation, deworming, and for SAM children, RUTF, amoxicillin and others as recommended in national nutritional protocols (Additional file 1: Table S1).

Children were followed for 42 days. Any clinical or laboratory adverse event was reported by the investigator as described elsewhere [19]. Serious adverse events were reviewed by a Data Safety and Monitoring Board.

### Laboratory methods

Only capillary blood was collected from finger pricks. SD Bioline® HRP2 RDT (Gyeonggi-do, Republic of Korea) was used for screening of malaria parasitemia. Thick and thin blood films were performed at baseline, at 6, 12, 24, 36, 48, and 72, hours, and at day 7, and then weekly until day 42, or in case of malaria signs. All blood films were read by two microscopists blinded to the other reading, and a third reading was performed in case of discrepancy. Films were read using a 100× objective and considered negative after 200 microscopic fields were assessed. *P. falciparum* asexual forms were counted on the thick film against at least 200 leukocytes [22]. Parasite density was calculated assuming a leukocyte density of 8000/μL. The presence of gametocytes was assessed.

Hemoglobin concentration was determined using HemoCue HB 301®-Hemoglobin brand device (Ängelholm, Sweden) on days 0 and 28. Anemia was defined as a hemoglobin concentration < 10 g/dL and severe anemia as a concentration < 7 g/dL.

PCR genotyping of malaria parasites collected from filter papers at enrolment and at the day of treatment failure were performed in MRTC laboratory in Bamako by amplification of the merozoite surface protein 2 (MSP-2) gene [23] and the microsatellites CA1 and TA87 [24]. Outcomes were defined as recrudescent if at least one shared allele was found with all three markers tested and as reinfection if day 0 and day of failure alleles were different in any of the three markers tested [25].

### Pharmacokinetics

A population-based sparse sampling approach was used to limit the number of PK samples required per child and concerned 150 SAM and 150 non-SAM children [26]. For each child, five capillary blood samples (50 μL spotted on filter paper) were collected; first, at 6, 12, 24, 36, or 48 hours (randomly allocated), second at 60 hours, third at 72 hours, fourth at day 7, and fifth at day 14 or day 21 (randomly allocated) post treatment initiation. Lumefantrine concentrations were measured at the Division of Clinical Pharmacology, University of Cape Town, using liquid chromatography tandem mass spectrometry as described previously [19].

### Outcomes

The primary outcome was the proportion of patients having an adequate clinical and parasitological response (ACPR) on day 28, after PCR correction.

Secondary outcomes were the proportions of PCR-corrected ACPR on day 42, non PCR-corrected ACPR, early therapeutic failure, late clinical failure, late parasitological failure on days 28 and 42 [8], proportion of reinfection and recrudescence, gametocyte carriage, hematological recovery as witnessed by hemoglobin change between baseline and day 28, and parasite clearance slope half-life.

The main PK outcome was lumefantrine concentration on day 7 since it is strongly correlated with the overall drug exposure in the terminal phase and therefore considered a good predictor of therapeutic response [27]. Secondary PK outcomes were measured lumefantrine concentrations at 60 and 72 hours post treatment initiation. Population-based PK modelling will be reported elsewhere.

### Statistical analysis

Unbalanced groups with the non-SAM/SAM ratio set to two was chosen both for ethical and practical reasons, because, for a fixed number of SAM children, twice the number of non-SAM allowed obtaining a higher power than a balanced design. A total of 540 children (180 SAM and 360 non-SAM) allowed detection of a minimum difference of 8 % (87 % ACPR in SAM vs. 95 % in non-SAM children), with a power of 80 %, two-sided significance level of 5 %, and taking into account up to 15 % dropouts. We planned to enroll two thirds of the sample in Mali during the 2013 and 2014 malaria seasons, and one third in Niger during the 2014 malaria season.

Study data were double entered using REDCap electronic data capture tools hosted at Epicentre [28], and analysis was performed with STATA 13, StataCorp®, College Station, TX, USA.

Analyses of treatment response were performed on two different populations: (1) modified intention-to-treat (mITT) population that included all enrolled patients with parasitological confirmation of mono-infection with *P. falciparum* with density > 1000/μL at screening, who took at least one dose of study drug; and (2) per protocol population including all patients who were part of the mITT and who completed the 3-day treatment course, did not experience major deviation, nor premature discontinuation before day 28 for other reason than failure. Safety analysis was performed in all patients who had received at least one dose of the study drug.

Comparisons of the main treatment outcomes (PCR-uncorrected and corrected ACPR, reinfection) were performed using two analysis methods: Kaplan–Meier analysis comparing the cumulative success rates and allowing to account for censored data, and simple comparison of proportions. The 95 % CIs were estimated using either Wald CI (for Kaplan–Meier estimators) or binomial exact CI (for proportions). Log-rank test for equality of survivor functions was used for comparison of survival curves. Comparisons of proportions were done using a χ^2^ or Fisher exact test.

For other outcomes (hematological recovery, gametocyte carriage, parasite clearance slope half-life), comparisons were performed between the SAM and non-SAM groups using a Student or Wilcoxon test for continuous variables and a χ^2^ or Fisher exact test for categorical variables. To calculate the parasite clearance slope half-life, the log-transformed parasite counts over time were modelled using the Parasite Clearance Estimator Tool developed by the WWARN [29].

Cox multivariable modelling investigated the effect of SAM and other cofactors (study site, baseline parasite density, child’s age, and all covariates with a statistically significant difference at baseline between the SAM and non-SAM groups) on malaria-free survival.

Finally, we compared lumefantrine concentration at 60 and 72 hours and at day 7 between groups using Wilcoxon rank-sum test, and we investigated if a lower day 7 lumefantrine concentration was associated with the risk of malaria infection using Cox modelling as described above.

Each adverse event was coded to a “Preferred Term” using the Medical Dictionary for Regulatory Activities, version 11 [30]. Then, the number and percentage of patients with at least one adverse event of the following categories were provided: those leading to treatment discontinuation, serious adverse events, and most common adverse events (≥5 %, regardless of the treatment group).

All analyses described above were also conducted after adjusting for study site, and site by site where the sample size allowed.

## Results

### Patient disposition and baseline characteristics

Overall, 871 children were assessed for eligibility, and 399 were included in the study. Respectively, 360 were enrolled in Mali (Nov 2013 to Jan 2014 then June to Dec 2014) and 39 children in Niger (Oct 2014 to Jan 2015), making a total of 399. Recruitment in Niger did not reach the targeted 120 children due to external constraints in the study site that have delayed the start of the inclusions. Following a Data Safety and Monitoring Board meeting held in February 2015, i.e., at the end of the planned recruitment period, the study was terminated before completion of the 540 inclusions, because efficacy results already obtained for the 218 first recruited children did not show any difference between groups or lack of efficacy (these interim results were in line with the final results which will be developed hereunder).

**Fig. 1:**
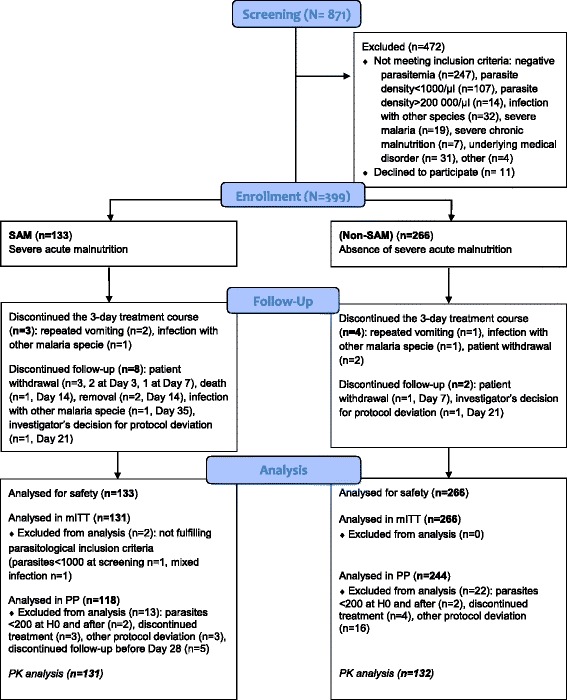
Study profile. SAM, severe acute malnutrition, mITT, modified intent-to-treat, PP, per protocol, PK, pharmacokinetics

Among the 133 and 266 children included in the SAM and non-SAM groups131 (98.5 %) and 266 (100 %), respectively, were part of the mITT population. After exclusion of patients with premature discontinuation or protocol deviations, 118 SAM (88.7 %) and 244 non-SAM (91.7 %) patients were included in the per protocol population (Fig. 1).

Apart from the anthropometric characteristics, which were de facto different between groups, SAM children were significantly younger than non-SAM (mean 17 vs. 28 months, *P* < 0.0001; Table 1), with only 10 % of SAM being older than 26 months in comparison to 50 % in the non-SAM group. Clinical presentation also differed with more frequent fatigue, anorexia, and diarrhea at onset in SAM children.

**Table 1:** Baseline characteristics by nutritional status, modified intent-to-treat population (N = 397)

	SAM (N = 131)	Non-SAM (N = 266)	*P*
Sociodemographic characteristics			
Season at inclusion, n (%)			0.943
High transmission (June–October)	66 (50.4)	133 (50.0)	
Low transmission (November–January)	65 (49.6)	133 (50.0)	
Age in months, mean (SD)	16.9 (7.7)	28.2 (13.0)	<0.0001
Male gender n (%)	65 (49.6)	122(45.9)	0.481
Rural residence, n (%)	126 (96.2)	260 (97.7)	0.373
Education of mother, n (%)			0.051
None	114 (87.0)	210 (78.9)	
Primary or secondary	17 (13.0)	56 (21.1)	
Has a mosquito net	107 (81.7)	215 (80.8)	0.838
In good state	47 (35.9)	92 (34.6)	0.648
Used it all nights the previous week	95 (72.5)	200 (75.2)	0.567
Anthropometric characteristics			
Weight in kg, mean (SD)	6.9 (1.1)	10.6 (2.5)	<0.0001
Height in cm, mean (SD)	74.4 (6.8)	84.5 (10.3)	<0.0001
Weight-for-height z-score, mean (SD)	−3.42 (0.55)	−1.01 (0.92)	<0.0001
MUAC in mm, mean (SD)	116.8 (6.6)	139.2 (12.1)	<0.0001
Weight-for-age z-score, mean (SD)	−3.39 (0.58)	−1.41 (0.93)	<0.0001
Stunting (height-for-age z-score < –2), n (%)	62 (47.3)	71 (26.7)	<0.0001
Clinical characteristics			
Measured temperature > 37.5 °C, n (%)	115 (89.2)	226 (85.6)	0.331
Fatigue, n (%)	112 (85.5)	198 (74.4)	0.012
Anorexia, n (%)	110 (84.0)	178 (66.9)	<0.0001
Vomiting, n (%)	27 (20.6)	58 (21.8)	0.785
Diarrhea, n (%)	37 (28.2)	21 (7.9)	<0.0001
Cough or bronchitis, n (%)	55 (42.0)	87 (32.7)	0.070
ENT (otitis media, rhinorrhea), n (%)	52 (39.7)	113 (42.5)	0.596
Splenomegaly, n (%)	25 (19.1)	46 (17.3)	0.662
Biological characteristics			
Parasite density (parasites/μL), median	10880	11520	0.7648
IQR	3683–32160	4200–39240	
Presence of gametocytes	40 (30.5)	63 (23.7)	0.143
Hemoglobin concentration (g/dL), mean (SD)	8.6 (1.5)	8.8 (1.5)	0.196
Hemoglobin <10 g/dL, n (%)*	104 (79.4)	196 (73.7)	0.214
Hemoglobin <7 g/dL, n (%)	20 (15.3)	32 (12.0)	0.369

*SAM* severe acute malnutrition, *SD* standard deviation, *MUAC* mid-upper arm circumference, *ENT*, ear nose and throat, *IQR* interquartile range

Baseline characteristics by study site are shown in Additional file 2: Table S2. Season at inclusion, type of habitat (rural vs. urban) baseline parasitemia, baseline hemoglobin, and mosquito net use were significantly different between sites.

### Treatment administration

Due to lower weight in children with SAM, the mean dose-weight received in the SAM group was significantly higher than in the non-SAM group, and frequently exceeded 100 mg/kg in SAM children (Table 2).

**Table 2:** Treatment administration, modified intent-to-treat population (N = 397)

	SAM N = 131	Non-SAM N = 266	*P*
Dose planned, n (%)			<0.0001
<15 kg: 20/120 mg, 1 tablet per intake, 6 tablets, n (%)	131 (100)	252 (94.7)	
≥15 kg: 20/120 mg, 2 tablets per intake, 12 tablets, n (%)	0	14 (5.3)	
Lumefantrine dose-weight (mg/kg) – mean (SD)	105.7 (18.6)	73.2 (18.1)	<0.0001
< 60 mg/kg (theoric efficacy threshold), n (%)	2 (1.6)	70 (26.5)	<0.0001
> 100 mg/kg (theoric toxicity threshold), n (%)	81 (62.8)	18 (6.8)	<0.0001
Early vomiting within 30 minutes after intake	49 (37.4)	55 (20.7)	<0.0001
Did not receive the total treatment dose^a^	3 (2.3)	5 (1.9)	0.784

^a^Seven children (three SAM and four non-SAM) discontinued the study before completing the 3-day treatment course: repeated vomiting, 2 in SAM and 1 in non-SAM; infection with other malaria species, 1 in SAM and 1 in non-SAM; patient withdrawal, 2 in non-SAM. For one non-SAM child, an error in administration caused him to receive 11 tablets overall instead of 12

*SAM* severe acute malnutrition

Early vomiting within half an hour after dosing was more frequent in SAM children (37.4 % vs. 20.7 %), and its frequency doubled when the dose-weight exceeded the toxicity threshold of 100 mg/kg [18]: (44/100 (44 %) of patients receiving more than 100 mg/kg reported vomiting vs. 60/297 (20.2 %) for those receiving a lower dose-weight, *P* < 0.0001).

### Treatment response

During the 42-day follow-up, 99 patients became parasitemic with *P. falciparum* at blood smear. After PCR correction, only four infections were classified as recrudescence.

In simple comparison of proportions, PCR-corrected ACPR at day 28 was 100 % (95 % CI, 96.8–100 %) in SAM versus 98.8 % (95 % CI, 96.4–99.7 %) in non-SAM (*P* = 0.236) patients. These results were consistent with those obtained by Kaplan–Meier analysis (Table 3).

**Table 3:** Kaplan–Meier estimates of primary and main secondary outcomes by nutritional status

	SAM		Non-SAM		
	n/N^a^	Kaplan–Meier estimate (95 % CI)	n/N^a^	Kaplan–Meier estimate (95 % CI)	*P*
Day 28 PCR-corrected ACPR^b^					
mITT	131/131	100 % (NA)	263/266	98.9 % (96.5–99.6)	0.232
PP	118/118	100 % (NA)	241/244	98.9 % (96.2–99.6)	0.227
Day 42 PCR-corrected ACPR					
mITT	131/131	100 % (NA)	262/266	98.3 % (95.6–99.4)	0.168
PP	118/118	100 % (NA)	240/244	98.2 % (95.3–99.3)	0.162
Day 28 uncorrected ACPR					
mITT	108/131	81.3 % (73.3–87.2)	223/266	83.5 % (78.4–87.5)	0.611
PP	96/118	81.3 % (73.0–87.3)	203/244	83.2 % (77.9–87.3)	0.668
Day 42 uncorrected ACPR					
mITT	102/131	76.4 % (67.9–83.0)	196/266	73.0 % (67.2–78.0)	0.552
PP	91/118	77.1 % (68.4–83.7)	176/244	72.1 % (66.0–77.3)	0.377
Day 28 reinfection					
mITT	23/131	18.7 % (12.8–26.8)	39/266	15.2 % (11.3–20.1)	0.374
PP	22/118	18.6 % (12.7–26.9)	37/244	15.3 % (11.4–20.5)	0.412
Day 42 reinfection					
mITT	29/131	23.6 % (17.0–32.1)	65/266	25.4 % (20.5–31.2)	0.811
PP	27/118	22.9 % (16.3–31.6)	63/244	26.2 % (21.1–32.3)	0.600

*P* values were calculated with log-rank test

^a^N, total number; n, number with event (adequate response or reinfection)

^b^Primary endpoint

*SAM* severe acute malnutrition, *ACPR* adequate clinical and parasitological response, *mITT* modified intent-to-treat population (N = 397), *PP* per protocol population (N = 362), *CI* Wald confidence interval, *NA* not assessable

PCR-corrected ACPR by day 42 as well as uncorrected ACPR by days 28 and 42 were not different between groups either (Table 3). The day-28 reinfection rate was 18.7 % (95 % CI, 12.8–26.8 %) in SAM versus 15.2 % (11.3–20.1 %) in non-SAM in mITT (*P* = 0.393). At day 42, it was 23.6 % (17.0–32.1 %) in SAM and 25.4 % (20.5–31.2 %) in non-SAM (*P* = 0.811).

The proportions of early therapeutic failure, late parasitological failure, and late clinical failure were similar in both groups (Additional file 3: Table S3).

The median parasite clearance slope half-life was 3.16 hours (interquartile range (IQR): 2.54–3.88) in SAM and 3.06 hours (IQR: 2.56–3.90) in non-SAM patients (*P* = 0.5655).

At baseline, 40/131 (30.5 %) of SAM versus 63/266 (23.7 %) of non-SAM patients had gametocytes in their blood (*P* = 0.14); these figures were 9.9 % versus 8.7 % (*P* = 0.68), respectively, at day 7, and 3.8 % versus 4.5 % (*P* = 0.75) at day 14, indicating no difference in gametocyte carriage between groups.

Hemoglobin at day 28 was available in 95 SAM and 212 non-SAM children. The mean change in hemoglobin level was 1.7 g/dL (from 8.7 at baseline to 10.4 at day 28) in SAM versus 2.1 g/dL (from 8.8 to 10.9) in non-SAM patients (*P* = 0.0372) indicating better hematological recovery in non-SAM children. This association remained significant after adjustment for study site, age and baseline parasitemia.

### Multivariable analysis of factors associated with reinfection

**Fig. 2:**
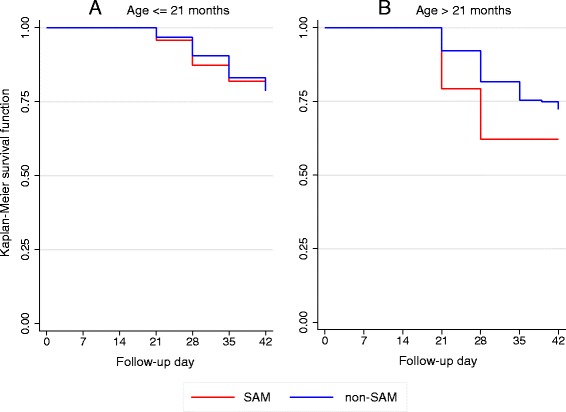
Kaplan–Meier curves of reinfection-free survival until day 42 in severe acute malnutrition (SAM) and non-SAM children, stratified by age. **a** Children ≤ 21 months. **b** Children > 21 months. Results are displayed for the modified intent-to-treat population (N = 397). Age strata are defined by the median value of 21 months. The log-rank tests for equality of survival functions are displayed hereafter: **a** *P* = 0.4836 at day 28, *P* = 0.7845 at day 42. **b** *P* = 0.0131 at day 28, *P* = 0.1684 at day 42

In univariable analysis, SAM was not associated with reinfection (Table 3). Due to the strong positive association of age with the risk of reinfection, and a marked heterogeneity in age distribution between SAM and non-SAM children, we categorized children by age strata, using 21 months (the median value in our total sample) as cut-off. Reinfection was twice more frequent in children > 21 months (21.5 % vs. 11.2 %, *P* = 0.007) and when considering these older children, SAM was associated with a two-fold higher hazard or reinfection in crude as well as adjusted analyses (adjusted hazard ratio (HR) = 2.10; 95 % CI, 1.05–4.22; *P* = 0.038; Table 4). However, we did not see such an association in the younger age stratum. The different risk of reinfection between arms was not observed after day 28 in any of the age strata as illustrated by the Kaplan–Meier survival curves being spaced apart on days 21 and 28 then closer on days 35 and 42 (Fig. 2b). *P* values for the log-rank test in the older age stratum were 0.0131 and 0.1684, respectively, at days 28 and 42.

**Table 4:** Cox univariable and multivariable modelling of factors associated with risk of reinfection by day 28 in the modified intent-to-treat population (N = 397, 62 reinfections)

		Univariable		Multivariable	
Variable	n/N^a^ (%)	HR (95 % CI)	*P*	HR (95 % CI)	*P*
Severe malnutrition by age group			0.0043		0.0111
≤ 21 months					
Non-SAM	9/98 (9.2)	0.50 (0.24–1.06)		0.51 (0.24–1.07)	
SAM	12/99 (12.1)	0.68 (0.35–1.33)		0.74 (0.38–1.45)	
> 21 months					
Non-SAM	30/168 (17.9)	1 (Ref)		1 (Ref)	
SAM	11/32 (34.4)	2.25 (1.12–4.48)		2.10 (1.05–4.22)	
Study site			0.054		0.244
Mali	61/359 (17.0)	1 (Ref)		1 (Ref)	
Niger	1/38 (2.6)	0.14 (0.02–1.04)		0.31 (0.04–2.25)	
Parasite density at inclusion (parasites/μL), per 1 log_10_ increase	28520 vs. 10200^b^	1.22 (1.03–1.45)	0.019	1.10 (0.95–1.27)	0.189
Season at inclusion			<0.0001		<0.0001
Nov–Jan (low)	5/198 (2.5)	1 (Ref)		1 (Ref)	
Jun–Oct (high)	57/199 (28.6)	12.43 (4.98–31.01)		10.27 (4.07–25.95)	
Has a mosquito net			0.047	___	NS^c^
No	18/75 (24.0)	1 (Ref)			
Yes	44/322 (13.7)	0.57 (0.33–0.99)			
Stunting (HFA < –2 z-score)			0.042	___	NS^c^
No	49/264 (18.6)	1 (Ref)			
Yes	13/133 (9.8)	0.53 (0.29–0.98)			
Lumefantrine dose-weight				___	NS^c^
> 60 mg/kg	45/324 (13.9)	1 (Ref)			
≤ 60 mg/kg	17/73 (23.3)	1.83 (1.05–3.20)	0.033		

Variables tested in univariable analysis were presence of severe acute malnutrition, age, study site, parasite density at inclusion, season at inclusion, sex, education level, type of residence, possession and use of a mosquito net, presence of stunting, moderate anemia, severe anemia, gametocytes at inclusion, early vomiting after treatment administration, and lumefantrine dose-weight received (artemether dose-weight was collinear with lumefantrine dose-weight as fixed combinations were used). Only variables with *P* < 0.10 are displayed here. Age, study site, and parasite density at inclusion were forced in multivariable models regardless of significance. Other variables were kept if *P* < 0.05

^a^N, total number; n, number with reinfection by day 28

^b^Median value in reinfected versus non-reinfected children are displayed

^c^Mosquito net possession, stunting, and lumefantrine dose-weight did not remain significantly associated with reinfection in multivariable analysis

*SAM* severe acute malnutrition, *HR* hazard ratio, *CI* Wald confidence interval, *Ref* reference, *HFA* height-for-age ratio, *NS* not significant

A complementary analysis was conducted in a subsample of 83 SAM and 83 non-SAM children who could be matched by age in months and study site, with age ranging from 6 to 50 months. In this subsample, SAM was associated with an increased risk of reinfection, with two-fold magnitude of risk (results shown in Additional file 4: Table S4).

### Lumefantrine concentration and its association with SAM and reinfection

**Fig. 3:**
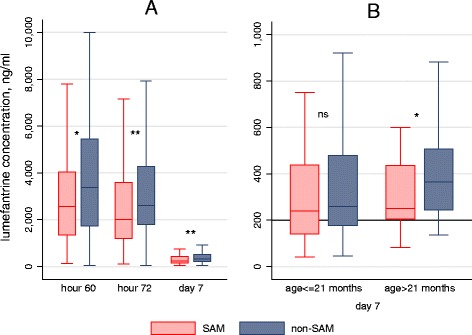
Boxplots of lumefantrine concentration by nutritional status. **a** On different time points. **b** On day 7 after stratification by age group. Boxplots show median and interquartile range, outside values are not shown. *SAM* severe acute malnutrition, *ns* not significant (for comparison between SAM and non-SAM). * *P* < 0.05; ** *P* < 0.01

Overall, 131 SAM and 132 non-SAM children were part of the PK cohort. Lumefantrine concentration was lower in younger (≤21 months) and SAM children on each analyzed time point. At day 7, the median was 241 ng/mL (IQR: 157–453) in younger (n = 140) versus 324 ng/mL (IQR: 227–503) in older children (n = 115; *P* = 0.0023). It was 246 ng/mL (IQR: 160–438) in SAM (n = 126) versus 330 ng/mL (IQR: 216–503) in non-SAM (n = 129; *P* = 0.0053; Fig. 3a). Interestingly, this was observed even though the total weight-dose received was higher in younger and SAM children. SAM was associated with a lower lumefantrine concentration on day 7 in the older children age stratum (mean 336 ng/mL in SAM vs. 405 ng/mL in non-SAM, *P* = 0.0498), but not in the younger one, in which both SAM and non-SAM had low concentrations (Fig. 3b). Of note, older SAM children were also those who experienced more reinfections. A day-7 concentration over 200 ng/mL (previously pointed by WWARN as an efficacy threshold) [31], was achieved in 57.1 % of younger SAM, 66.7 % of younger non-SAM, 75.0 % of older SAM, and 82.8 % of older non-SAM (*P* = 0.002 overall) patients. We did not find a statistically significant association between lumefantrine concentration on day 7 and protection from reinfection by day 28 (in Cox modelling, HR = 1.05, 95 % CI, 0.68–1.64, *P* = 0.818 for the measure of association between reinfection and log-transformed lumefantrine concentration in continuous variables, after adjusting for age).

No differences in any of the above results and conclusions regarding treatment efficacy and lumefantrine concentration were observed while adjusting for study site, or considering Mali data alone.

### Adverse events

The proportion of children who reported at least one adverse event was similar between SAM (112, 84.2 %) and non-SAM (221, 83.1 %; *P* = 0.775). Gastrointestinal disorders were more frequently reported in children with SAM (Table 5). Among the six serious adverse events (four in SAM, two in non-SAM, *P* = 0.09), five had a favorable outcome, whereas one (a case of meningitis in a SAM child, unconfirmed bacteriologically) led to death.

**Table 5:** Adverse events by preferred term and system organ class – safety population (N = 399)

System organ class	Preferred term	SAM (N = 133), n (%)	Non-SAM (N = 266), n (%)
Adverse events reported with frequency > 5 %			
Blood and lymphatic system disorders		6 (4.5)	19 (7.1)
	Anemia	6 (4.5)	19 (7.1)
Eye disorders		4 (3)	15 (5.6)
	Conjunctivitis	4 (3)	15 (5.6)
Gastrointestinal disorders^a^		53 (39.8)^a^	77 (28.9)^a^
	Abdominal distension^a^	10 (7.5)^a^	5 (1.9)^a^
	Diarrhea^a^	24 (18.0)^a^	46 (17.3)^a^
	Gastroenteritis^a^	15 (11.3)^a^	10 (3.8)^a^
	Vomiting	6 (4.5)	7 (2.6)
Respiratory, thoracic and mediastinal disorders		71 (53.4)	141 (53)
	Bronchitis	38 (28.6)	83 (31.2)
	Cough	11 (8.3)	16 (6)
	Nasopharyngitis	7 (5.3)	15 (5.6)
	Rhinorrhea	15 (11.3)	29 (10.9)
Serious adverse events			
Blood and lymphatic system disorders		1 (0.8)	1 (0.4)
	Anemia	1 (0.8)	1 (0.4)
Gastrointestinal disorders		0 (0)	1 (0.4)
	Gastrointestinal motility disorder	0 (0)	1 (0.4)
Nervous system disorders		1 (0.8)	0 (0)
	Meningitis bacterial^b^	1 (0.8)	0 (0)
Respiratory, thoracic and mediastinal disorders		1 (0.8)	0 (0)
	Lower respiratory tract infection	1 (0.8)	0 (0)
Infections and infestations		1 (0.8)	1 (0.4)
	Plasmodium falciparum infection^c^	1 (0.8)	1 (0.4)
Adverse events causing treatment discontinuation		2 (1.5)	1 (0.4)
Gastrointestinal disorders		2 (1.5)	1 (0.4)
	Vomiting^d^	2 (1.5)	1 (0.4)

Adverse events are reported in alphabetical order of system organ class, then preferred term, according to the MedDRA dictionary (Medical Dictionary for Regulatory Activities, version 11)

^a^Statistically significant difference between SAM and non-SAM (more gastro-intestinal disorders in SAM)

^b^Caused death

^c^Severe malaria in an HIV-coinfected patient

^d^In all cases, children vomited less than 30 minutes after drug intake and had iterative vomiting on re-administration

*SAM* severe acute malnutrition

## Discussion

This study was the first to assess AL efficacy in SAM compared to non-SAM children and showed that the lower bound of PCR-corrected ACPR was superior to 90 % (96.8 % and 96.4 %, respectively) in both groups, indicating adequate therapeutic efficacy. This similar treatment response suggests that AL efficacy would not be impaired in SAM children, when these children receive RUTF concomitantly. Moreover, no early treatment failure and no difference in parasite clearance were observed, showing that the efficacy of the artemisinin component is also maintained in SAM children.

The only previously published longitudinal study assessing AL efficacy in malnourished children was conducted in stunted and underweight children and also concluded that AL had satisfactory efficacy [16]. However, the WWARN found that the risk of treatment failure with AL was greatest in children underweight-for-age (i.e., suffering from global malnutrition) aged 1–3 years in Africa [18]. In our study, AL was administered concomitantly with RUTF in SAM children, which might have contributed to an improved drug absorption in this group. These results of a reassuring efficacy profile might not be extrapolated to SAM children who are not captured by nutritional rehabilitation programs, thus not receiving RUTF nor will they apply for SAM children with complications needing inpatient care, although these children are eligible for IV artesunate treatment instead of oral ACTs.

Due to the well-known, major effect of age on malaria susceptibility and a marked difference in age between SAM and non-SAM children, we further conducted age-stratified analyses. In older children (>21 months), where reinfections were more frequent than in younger children, SAM was associated with a two-fold higher risk of reinfection by day 28. A complementary analysis in a subsample of SAM and non-SAM children matched by age retrieved similar HRs around 2, suggesting that SAM would be associated with an increased risk of reinfection until day 28 regardless of the confounding effect of age. The limited duration of this higher-risk period (which did not remain at day 42) is compatible with a lack of post-treatment prophylactic effect of AL after 28 days [32]. The significantly slower hematological recovery in SAM children is probably mostly due to the effect of nutritional deficiencies on hematopoiesis; this difference was observed considering that all SAM and a majority of non-SAM children received iron and folic acid supplementation.

We observed a lower day 7 lumefantrine concentration in older SAM (who experienced more reinfections) than in older non-SAM children, but it was similar to what was measured in younger SAM and non-SAM (who altogether experienced less reinfections). A recent meta-analysis conducted by WWARN also pointed out that day 7 lumefantrine concentration was lower in younger and underweight children, though it did not investigate the effect of SAM specifically [31].

However, we were not able to show a statistically significant association between day 7 lumefantrine concentration and the risk of reinfection by day 28, neither in the overall population nor in older children, although the latter sub-group analysis probably lacked power. Age acts as a strong confounder in investigating this association. It has been negatively associated with lumefantrine concentration in this study and others [31, 33], and children < 3 years have usually lower exposure to lumefantrine [15]. Additionally, unmeasured factors linked to age modulate the exposition and susceptibility to malaria: different exposure to mosquito bites in relation to behavioral changes in older children possibly mediated by different utilization of insecticide-treated nets [34], and different immune premunition switching from transmitted maternal immunity via breastfeeding to acquired host immunity.

We confirmed here that the relation between the dose-weight received and the lumefantrine concentration measured in blood is not straightforward, the higher dose-weight received in younger SAM children resulted in a lower day 7 concentration [31]. Though not correlated with a higher risk of treatment failure or reinfection in our study, the low concentration achieved in SAM and young children is still a concern, as sub-optimal exposure to antimalarials is the first step to the emergence of parasite resistance [35]. The question of adapting dosing strategies in younger and malnourished children should be investigated further [18]. The ongoing PK modelling of our data will include simulation of different AL dosing scenarios to assess their effect on lumefantrine PK profile. Additional studies will also be needed before recommending a change in dosing in SAM children considering the safety profile as well as the observations confirmed herein that vomiting was more frequent when the dose-weight exceeded 100 mg/kg (as pointed by WWARN earlier) [18]. Gastrointestinal toxicity is a major concern in children with uncomplicated SAM because it can compromise outpatient refeeding with RUTF. It is also likely to affect therapeutic efficacy outside of the frame of a clinical trial, as the drug would not always be given again after vomiting.

PK modelling will also investigate whether SAM is associated with decreased absorption and/or increased volume of distribution of lumefantrine in relation to age and other confounders. We noted that younger children with SAM had more frequent early vomiting after treatment intake. The drug was re-administered within 30 minutes, so the lower concentration is unlikely to be caused by the vomiting of the first dose. However, an indirect effect (empty stomach, change in gastric pH, vomiting of RUTF) could have impaired the drug absorption. Moreover, absorption of lumefantrine is known to be dose-limited with saturation at higher dose [36], so the part of the first dose that was not vomited might have contributed to saturate the absorption of the second dose.

Our study has several limitations. First, we did not match recruitment on age in SAM and non-SAM groups. SAM children were younger than non-SAM because the peak of prevalence of malnutrition is observed at earlier ages than that of malaria. We took the effect of age into account in age-stratified analyses and age-matched subgroup analyses, but it led to a lack of power. Future studies should match recruitment on age or recruit preferentially older SAM children to deal with the confounding, prominent effect of age in the comparison between SAM and non-SAM children. Another confounding factor could be the use of RUTF in SAM children, which could have contributed to improved absorption in this group compared to the other arm where AL was administered with milk; other associated therapies, such as iron, folic acid, and albendazole, were given to SAM as well as non-SAM children. Amoxicillin was administered to SAM children only, though there is no evidence of metabolism interactions between amoxicillin and AL. Finally, the recruitment obtained in Niger was far below our expectations. Nevertheless, the power reached with our sample size was 81 % to see a difference of 8 % around the observed 98 %, so we are confident that this difference between groups truly did not exist. Although limited, Niger data were generally consistent with Mali data. The important difference in reinfection rates between sites was linked to the different recruitment periods, as we only captured the end of the malaria season in Niger (inclusions between end of October 2014 and January 2015). Adjusting for the study site or considering only Mali data in analyses led to very similar results as in the overall population. There is no reason to believe that physiopathologic mechanisms determining the PK profile of ACTs in SAM would be different in Mali and Niger or other sub-Saharan African countries.

## Conclusions

This study is the first to have compared the efficacy of AL in SAM versus non-SAM children in relation to drug concentration, using a comparative intervention design. It showed comparable therapeutic efficacy of AL given concomitantly with RUTF in children with SAM compared to non-malnourished children, although a lower day 7 lumefantrine concentration in SAM children could impact its post-treatment prophylactic effect, as witnessed by more frequent reinfections until day 28.

Sub-therapeutic concentrations of a drug do not necessarily translate into higher failure at individual level but would undoubtedly contribute to selecting resistant parasites and increasing the risk of treatment failure at population level. We believe further studies should urgently answer the question of whether ACT dosing strategies are adequate in children with SAM.

## Box 1. Eligibility criteria

Inclusion criteria:

- Age between 6 and 59 months
- Weight ≥ 5 kg
- Axillary temperature ≥ 37.5 °C or history of fever during the previous 24 hours as reported by the parent/guardian
- *P. falciparum* monoinfection confirmed on blood film
- Parasitic density between 1000 and 200,000 asexual forms/μL of blood
- High probability of compliance with follow-up visits (no near-term travel plans)
- Written consent of a parent or guardian who is at least 18 years of age
- According to the group: in SAM children, weight-for-height z-score < –3 SD or MUAC < 115 mm and/or bilateral edema, and in non-SAM children, weight-for-height z-score ≥ –3 SD, and MUAC ≥ 115 mm

Exclusion criteria:

- General danger signs or signs of severe malaria as defined by the World Health Organization
- Mixed or mono-infection with another *Plasmodium* species detected by microscopy
- Severe anemia (hemoglobin <5 g/dL)
- Known underlying chronic or severe disease (e.g., HIV/AIDS, TB, cardiac, renal or hepatic disease, sickle cell)
- Presence of febrile conditions due to diseases other than malaria which could alter the outcome of the study
- Known history of hypersensitivity or contra-indication to any of the study medications: artemether, lumefantrine (first-line medications), or artesunate, amodiaquine (rescue medications). History of a full treatment course with artemether-lumefantrine in the past 14 days
- Height-for-age < –3 z-score (severe chronic malnutrition)
- Severe complications of malnutrition requiring hospitalization in intensive care or stabilization, including kwashiorkor

## Additional files

Additional file 1: Table S1. Concomitant medications and rescue medications administered in the Mal-Nut study. (DOCX 18 kb)
Additional file 2: Table S2. Baseline characteristics by study site, modified intent-to-treat population (N = 397). (DOCX 19 kb)
Additional file 3: Table S3. Types of treatment failure by nutritional status. (DOCX 18 kb)
Additional file 4: Table S4. Cox univariate and multivariate modelling of factors associated with risk of reinfection by day 28 in the subsample of children matched by age (N = 166, 83 SAM and 83 non-SAM, 26 reinfections). (DOCX 20 kb)

## Abbreviations

ACPR: adequate clinical and parasitological response
ACTs: artemisinin-based combination therapies
AL: artemether-lumefantrine
CI: confidence interval
HR: hazard ratio
IQR: interquartile range
mITT: modified intention-to-treat
MUAC: mid-upper arm circumference
PK: pharmacokinetic
RUTF: ready-to-use therapeutic food
SAM: severe acute malnutrition
SD: standard deviation
WWARN: WorldWide Antimalarial Resistance Network
