# Supplementary material for: Efficacy of artemether-lumefantrine in relation to drug exposure in children with and without severe acute malnutrition: an open comparative intervention study in Mali and Niger
Source: BMC Med. 2016 Oct 24;14:167. doi: 10.1186/s12916-016-0716-1 (PMC5079061; doi:10.1186/s12916-016-0716-1)
Supplement: Additional file 1: Table S1. — Concomitant medications and rescue medications administered in the Mal-Nut study. (DOCX 18 kb) [file 12916_2016_716_MOESM1_ESM.docx]

**Additional file 1: Table S1. Concomitant medications and rescue medications administered in the Mal-Nut study.**

| **Medication** | **Dose** | **Timing/Duration** | **Condition** |
| --- | --- | --- | --- |
| Concomitant medications |  |  |  |
| Albendazole | 400 mg | Day 14 | All children |
| Folic Acid* | 5 mg | Enrolment | All children |
| Iron supplementation* | 50 to 200 mg daily | Until recovery | Children with anemia<10 g/dl |
| RUTF: Plumpynut® | 170 kcal / kg / day | From enrolment until nutritional recovery | SAM children |
| Amoxicillin | 50 to 100 mg/kg/day | 7 days from enrolment | SAM children |
| Vitamin A | 100 000 UI < 12 months  200 000 UI ≥12 months | Day 28 | SAM children who did not receive supplementation during the past 4 months |
| Measles vaccination | -- | Day 28 | Unvaccinated SAM children |
| Rescue medications |  |  |  |
| Artesunate-Amodiaquine, ASAQ Winthrop®, 25 mg/67.5 mg | 1 tablet daily ≤ 9 kg  2 tablets daily > 9 kg | 3 days | treatment failure, repeated vomiting or malaria infection with species other than *P. falciparum* |
| Artesunate | 2.4 mg/Kg IV H0, H12, then daily | Until oral route possible | Same as above and oral route impossible, or severe malaria |

* Iron and folic acid supplementations were included in RUTF thus systematic in SAM children (daily dose included in RUTF was 23 to 46 mg iron and 400 to 800 µg folic acid).
