# Supplementary material for: Efficacy of artemether-lumefantrine in relation to drug exposure in children with and without severe acute malnutrition: an open comparative intervention study in Mali and Niger
Source: BMC Med. 2016 Oct 24;14:167. doi: 10.1186/s12916-016-0716-1 (PMC5079061; doi:10.1186/s12916-016-0716-1)
Supplement: Additional file 2: Table S2. — Baseline characteristics by study site, modified intent-to-treat population (N = 397). (DOCX 19 kb) [file 12916_2016_716_MOESM2_ESM.docx]

**Additional file 2: Table S2. Baseline characteristics by study site, modified intent-to-treat population (N=397).**

|  | **Mali (N=359)** | **Niger (N=38)** | **P** |
| --- | --- | --- | --- |
| **Socio-demographic characteristics** |  |  |  |
| Season at inclusion – n (%)  High transmission (June-October)  Low transmission (November-January) | 193 (53.8%)  166 (46.2%) | 6 (15.8%)  32 (84.2%) | **<0.0001** |
| Age in months - mean (SD) | 24.2 (12.7) | 26.2 (13.1) | 0.3438 |
| Male gender n (%) | 165 (46.0%) | 22(57.9%) | 0.175 |
| Rural residence – n (%) | 355 (98.9%) | 31 (81.6%) | **<0.0001** |
| Education of mother -n (%)  None  Primary or secondary | 289 (80.5%)  70 (19.5%) | 35 (92.1%)  3 (7.9%) | 0.120 |
| Has a mosquito net  In good state  Used it all nights the previous week | 284 (79.1%)  114 (31.8%)  259 (72.1%) | 38 (100%)  25 (65.8%)  36 (94.7%) | **<0.0001**  **<0.0001**  **0.001** |
| **Anthropometric characteristics** |  |  |  |
| Weight in kg - mean (SD) | 9.4 (2.8) | 9.2 (2.5) | 0.8091 |
| Height in cm -mean (SD) | 81.3 (10.5) | 80.4 (9.2) | 0.8672 |
| Weight-for-Height z-score -mean (SD) | -1.82 (1.42) | -1.87 (1.30) | 0.8178 |
| MUAC in mm -mean (SD) | 132.0 (15.0) | 129.2 (15.2) | 0.3019 |
| Weight-for-Age z-score - mean (SD) | -2.02 (1.26) | -2.50 (1.08) | **0.0316** |
| Stunting (Height-for-Age z-score<-2) – n (%) | 110 (30.6%) | 23 (60.5%) | **<0.0001** |
| **Clinical characteristics** |  |  |  |
| Measured temperature > 37.5 °C -n (%) | 313 (87.2%) | 31 (81.6%) | 0.320 |
| Fatigue -n (%) | 310 (86.4%) | 0 | **<0.0001** |
| Anorexia -n (%) | 288 (80.2%) | 0 | **<0.0001** |
| Vomiting -n (%) | 75 (20.9%) | 10 (26.3%) | 0.413 |
| Diarrhea -n (%) | 43 (12.0%) | 15 (39.5%) | **<0.0001** |
| Cough or bronchitis -n (%) | 131 (36.5%) | 11 (29.0%) | 0.381 |
| ENT (otitis media, rhinorrhea) -n (%) | 156 (43.5%) | 9 (23.7%) | **0.024** |
| Splenomegaly-n (%) | 66 (18.4%) | 5 (13.2%) | 0.511 |
| **Biological characteristics** |  |  |  |
| Parasite density (parasites/microliter) – median [IQR] | 10600  [3920-35720] | 20139  [7446-54720] | **0.0466** |
| Presence of gametocytes | 97 (27.0%) | 6 (15.8%) | 0.173 |
| Hemoglobin concentration (g/dl) - mean (SD) | 8.6 (1.5) | 9.4 (1.4) | **0.0083** |
| Hemoglobin <7g/dl -n (%) | 51 (14.2%) | 1 (2.6%) | **0.043** |

NOTE. P values are calculated with Fisher exact or Wilcoxon rank-sum test.SD, standard deviation; MUAC, mid-upper arm circumference; ENT, ear nose and throat; IQR, interquartile range
