# Supplementary material for: Efficacy of artemether-lumefantrine in relation to drug exposure in children with and without severe acute malnutrition: an open comparative intervention study in Mali and Niger
Source: BMC Med. 2016 Oct 24;14:167. doi: 10.1186/s12916-016-0716-1 (PMC5079061; doi:10.1186/s12916-016-0716-1)
Supplement: Additional file 3: Table S3. — Types of treatment failure by nutritional status. (DOCX 18 kb) [file 12916_2016_716_MOESM3_ESM.docx]

**Additional file 3: Table S3. Types of treatment failure by nutritional status.**

|  | **SAM** | |  | **Non-SAM** | | **P** |
| --- | --- | --- | --- | --- | --- | --- |
|  | n/N* | Percentage (95% CI)† |  | n/N* | Percentage (95% CI)† |  |
| **Early therapeutic failure**  mITT  PP | 0/128  0/118 | 0% (0-2.8)†  0% (0-3.1)† |  | 0/262  0/244 | 0% (0-1.5)†  0% (0-1.4)† | NA  NA |
| **Late treatment failure at day 28** |  |  |  |  |  |  |
| Late clinical failure  mITT  PP | 5/123  5/118 | 4.1% (1.3-9.2)  4.2% (1.4-9.6) |  | 10/260  10/244 | 3.8% (1.9-7.0)  4.1% (2.0-7.4) | 0.918  0.820 |
| Late parasitological failure  mITT  PP | 18/123  17/118 | 14.6% (8.9-22.1)  14.4% (8.6-22.1) |  | 33/260  31/244 | 12.7% (8.9-17.4)  12.7% (8.8-17.5) | 0.510  0.330 |
| **Late treatment failure at day 42** |  |  |  |  |  |  |
| Late clinical failure  mITT  PP | 7/122  7/117 | 5.7% (2.3-11.5)  6.0% (2.4-11.9) |  | 16/258  16/242 | 6.2% (3.6-9.9)  6.6% (3.8-10.5) | 0.859  0.820 |
| Late parasitological failure  mITT  PP | 22/122,  20/117 | 18.0% (11.7-26.0)  17.1% (10.8-25.2) |  | 54/258  52/242 | 20.9% (16.1-26.4)  21.5% (16.5-27.2) | 0.510  0.330 |

Note – Types of failures displayed here follow the WHO classification for therapeutic response, and are not PCR-corrected. SAM, severe acute malnutrition; CI, confidence interval; mITT, modified intent-to-treat population; PP, per protocol population; NA, not assessable

*N, total number; n, number with failure. The total number is the number of patients assessable at the time point (day 3 for early failure, day 28 or 42 for late failure).

† Exact confidence interval is displayed. When no failure is observed, 97.5% unilateral confidence interval is displayed.
