# Supplementary material for: Efficacy of artemether-lumefantrine in relation to drug exposure in children with and without severe acute malnutrition: an open comparative intervention study in Mali and Niger
Source: BMC Med. 2016 Oct 24;14:167. doi: 10.1186/s12916-016-0716-1 (PMC5079061; doi:10.1186/s12916-016-0716-1)
Supplement: Additional file 4: Table S4. — Cox univariate and multivariate modelling of factors associated with risk of reinfection by day 28 in the subsample of children matched by age (N = 166, 83 SAM and 83 non-SAM, 26 reinfections). (DOCX 20 kb) [file 12916_2016_716_MOESM4_ESM.docx]

**Additional file 4: Table S4. Cox univariate and multivariate modelling of factors associated with risk of reinfection by day 28 in the subsample of children matched by age (N=166, 83 SAM and 83 non-SAM, 26 reinfections).**

|  |  | **Univariate** | |  | **Multivariate** | |
| --- | --- | --- | --- | --- | --- | --- |
| **Variable** | **n/N* (%)** | **HR (95%CI)** | **P** |  | **HR (95%CI)** | **P** |
| Severe acute malnutrition  No  Yes | 9/83 (10.8%)  17/83 (20.5%) | 1 (Ref)  2.05 (0.91-4.59) | 0.082 |  | 1 (Ref)  2.27 (1.01-5.09) | **0.048** |
| Study site  Mali  Niger | 25/156 (16.0%)  1/10 (10.0%) | 1 (Ref)  0.59 (0.80-4.33) | 0.602 |  | 1 (Ref)  1.47 (0.19-11.35) | 0.711 |
| Parasite density at inclusion (parasites/microliter), per 1 log_10_ increase | 25680 vs 8200† | 1.45 (1.11-1.90) | **0.006** |  | 1.26 (0.99-1.60) | 0.061 |
| Season at inclusion  Nov-Jan (low)  Jun-Oct (high) | 1/75 (1.3%)  25/91 (27.5%) | 1 (Ref)  23.56 (3.13-170.52) | **0.0002** |  | 1 (Ref)  22.80 (3.00-173.67) | **0.003** |

Note- The subsample included pairs of SAM and non-SAM children who could be matched by age in months and study site. If several children of the same age were available for matching, we chose to match the ones with the closer recruitment date. Variables tested in univariate analysis were those retained in the first step of modelling in the overall population (cf table 4): presence of severe acute malnutrition, age, study site, parasite density at inclusion, season at inclusion, possession of a mosquito net, presence of stunting, lumefantrine dose-weight received. Only variables with P<0.10 are displayed here. Study site and parasite density at inclusion were forced in multivariate models regardless of significance. Age was not forced because of the matching. Other variables were kept if P<0.05.

SAM, severe acute malnutrition; HR, hazard ratio; CI, Wald confidence interval; Ref, reference.

* N, total number; n, number with reinfection by day 28.

† median value in reinfected versus non reinfected are displayed
